# Treatment of Horizontal Root Fractures in Traumatized Maxillary Central Incisors Using Minimally Invasive Surgical and Prosthodontic Foundation Techniques

**DOI:** 10.1155/crid/9791300

**Published:** 2025-05-19

**Authors:** Katsuyuki Atsumi, Naomi Tanoue

**Affiliations:** ^1^Dental Clinic K, Kawaguchi, Saitama, Japan; ^2^Division of Developmental and Nurturing Dentistry, Nagasaki University Graduate School of Biomedical Sciences, Nagasaki, Japan

**Keywords:** foundation, glass fiber, periodontal surgery, resin cement, tooth fracture

## Abstract

**Objective:** The aim of this study is to present cases of root preservations in which minor surgery and characteristic multifiber foundation were performed to treat maxillary central incisor root fractures near the bone margin.

**Clinical Considerations:** Two patients experienced root fractures of the central incisors due to short- or long-term trauma. Orthodontic extrusion was not employed in both cases; instead, minimal periodontal surgery was performed as pretreatment. In cases where the patient's tooth remained intact, fractured pieces were bonded. However, in cases where the root of the tooth was fractured with the prosthesis, a new restoration was fabricated. The roots were constructed using multiple characteristically placed glass fiber posts and materials with high biocompatibility and hydrophilicity as the foundation. Fractured teeth were esthetically restored using conservative or prosthetic treatment methods, resulting in patient satisfaction. No complications were observed at the 4-year follow-up.

**Conclusions:** Although horizontal root fractures near the alveolar bone are generally considered to have a poor prognosis and esthetic outcome, they can be restored esthetically with minimal invasion by selecting appropriate procedures and materials.

**Summary**

• This case series demonstrates the effectiveness of combining minimally invasive surgeries and biocompatible materials in treating horizontal root fractures near the alveolar bone margin.
• The innovative use of multiple glass fiber–reinforced epoxy posts improves fracture resistance, ensuring long-term stability and esthetic outcomes, which are crucial in esthetic dentistry.

## 1. Introduction

Root fractures of permanent teeth reportedly occur in less than 8% of permanent dental trauma cases [1]. These fractures are broadly classified into horizontal and vertical root fractures. The indication for the treatment type depends primarily on the level of the fracture line [1], and fractured teeth present complex healing patterns. Treatment options range from simple observation and follow-up to conservative management or complex surgical procedures [2–4].

Horizontal root fractures of the central incisors due to trauma are rare, typically occurring in the middle third of the root and rarely in the apical third [5]. Moreover, favorable healing has been reported for horizontal root fractures at the apical third of a permanent maxillary central incisor [6]. Conversely, survival is poorest for root fractures in the gingival third of the root [7]. In particular, horizontal root fractures on the labial side, rather than the palatal side, require careful handling [8]. Furthermore, the absence of a ferrule is detrimental to the longevity of the fixed prosthesis [9].

Although various approaches to treating root fractures have been reported [2–4, 10, 11], the selection of the surgical procedure [12, 13] and dental materials [14] around the fracture area is assumed to play a significant role in the prognosis of the affected tooth. However, establishing an appropriate treatment plan for transversely fractured teeth near the alveolar bone margin is desirable owing to the poor prognosis for this condition.

Thus, in this case series, we aimed to describe the surgical and bonding procedures selected to preserve transversely fractured central incisors near the alveolar rim. The findings of the cases reported herein will contribute to improving oral health care, influencing policy, or guiding future research.

## 2. Case Presentation

The patients were given detailed information regarding the purpose of the report, the nature of the information to be shared, and the measures taken to protect their privacy and confidentiality. Consent was obtained from the patients for the publication of this report.

### 2.1. Case 1

**Figure 1:**
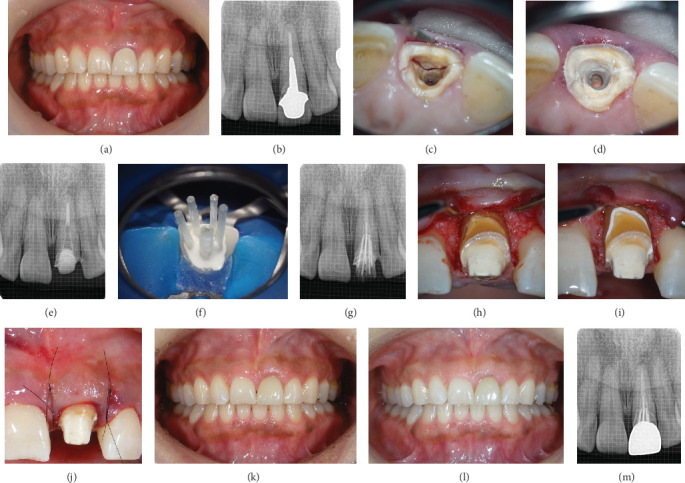
Case 1. (a) Frontal view at the first visit. (b) Radiograph of the anterior teeth taken on the day of the first visit. A translucent image is observed at the cervical area of the maxillary left central incisor. (c) Labial fracture line of the maxillary left central incisor after the removal of the post material. (d) The fractured piece bonded to the tooth root substance. (e) Radiograph after root canal treatment of the maxillary left central incisor. (f) Glass fiber posts distinctively positioned at the center (one main point) and cavity side (four subpoints). (g) Radiograph after foundation construction. (h) The fracture line is visible after opening the flap. (i) The fracture line is covered with MMA-based resin cement. (j) Periodontal tissue after surgery. (k) Frontal view after cementation of the suprastructure. (l) Frontal view 4 years after intraoral cementation. (m) Radiograph 4 years after intraoral cementation.

A 49-year-old female patient visited the author's dental clinic (Dental Clinic K) with a chief complaint of movement of the maxillary left central incisor (Figure 1a). Her chief complaint was mobility, not pain; however, she did report discomfort in the labial gingiva. The tooth had been restored with a metal-ceramic crown, and a dental periapical radiographic examination revealed no apical lesions and sufficient remaining bone volume; a long metal post was observed on the root of the tooth (Figure 1b). For standardizing dental radiographic imaging, film holders were used to maintain the correct angle and distance.

After the clinical examination, the cause of the movement was assumed to be a root fracture, and the post material was carefully removed. A transverse tooth root fracture was confirmed 8 mm below the gingival margin on the buccal side (Figure 1c). The depth of the fracture was assessed by carefully measuring the distance with a periodontal probe under a microscope. Even if the fracture was subgingival, it was possible to assess the condition from within the root canal.

As an alternative treatment, the extraction of the tooth and replacement using a fixed partial prosthesis or implant were considered; however, the patient strongly desired to preserve and restore the traumatized tooth and did not agree to any of the alternative treatment approaches. As a result, a treatment plan aiming to preserve the tooth was chosen. The fractured piece was bonded to the tooth root substance using methyl methacrylate (MMA)–based resin cement (Super-Bond Radiopaque, Sun Medical Company, Limited, Moriyama, Japan) [15–18] (Figure 1d). During bonding, moisture was controlled by air drying using a fine suction tip designed for root canal treatment. Thus, we were able to maintain complete dry conditions.  Figure 1e presents the dental radiographic image after completion of the root canal filling. The bonded, fractured piece served as a dental dam during the endodontic treatment. The foundation was restored using a glass fiber–reinforced epoxy post system (i-TFC Luminous, Sun Medical Co. Ltd.) and the resin cement. In addition to the main post, subglass fiber–reinforced posts were placed around the root canal wall to increase the strength of the tooth root [19, 20]. In this case, one main post (diameter: 1.6 mm) and four subposts (diameter: 1.0 mm each) were arranged (Figure 1f,g). After the foundation was complete, the provisional restoration was luted.

Thereafter, minor dental surgery was performed to obtain healthier periodontal tissues and improve esthetic outcomes. Opening the flap around the affected tooth revealed a vertical bone defect on the labial side, with the fracture also located on the bony margin (Figure 1h). Regenerative periodontal therapy was performed to reduce the periodontal pockets.

The fresh surface of the labial fracture was exposed and covered with MMA-based resin cement (Figure 1i). After removing the excess material, trafermin (REGROTH Dental Kit, Kaken Pharmaceutical Company, Limited, Tokyo, Japan) was applied for periodontal tissue regeneration. For protecting the blood coagulum, the wound was stabilized by means of an optimal flap design and adequate suturing techniques. The primary wound was healed with passive adaptation of the flap and complete wound closure (using appropriate suturing techniques) (Figure 1j). The provisional restoration was refabricated and securely fixed to both adjacent teeth using clear-colored MMA-based resin cement (Super-Bond Clear, Sun Medical Co. Ltd.).

The provisional restoration was followed up for 10 months after the treatment. After confirming the stabilization of the periodontal tissue, the final prosthetic treatment was performed using zirconia (Tanaka Enamel ZR Multi 5, ATD Japan Company, Limited, Tokyo, Japan) (Figure 1k).

The patient was followed up for 3 months after the treatment. Clinically acceptable periodontal and prosthetic conditions were achieved, and the patient was satisfied with the treatment outcomes 4 years after intraoral cementation (Figure 1l). The radiograph obtained 4 years after intraoral cementation revealed no abnormalities (Figure 1m).

### 2.2. Case 2

**Figure 2:**
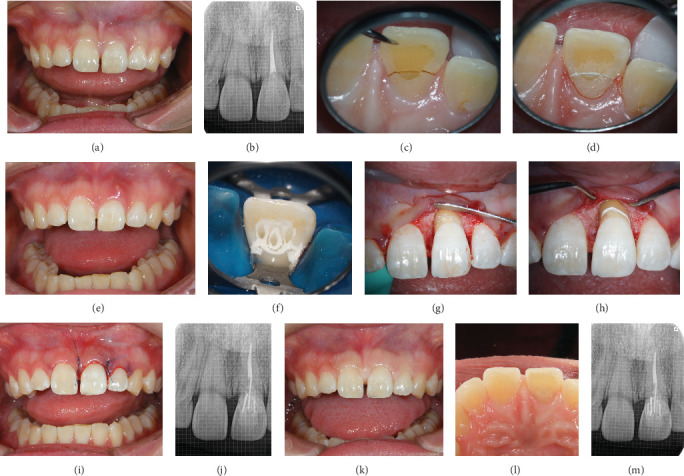
Case 2. (a) Frontal view of the maxillary anterior teeth at the first visit. (b) Radiograph of the anterior teeth at the first visit. (c) Palatal view of the fracture line of the maxillary left central incisor. (d) Palatal view after temporary fixation. (e) A sinus tract appeared on the labial gingiva. (f) Fixation using multiple fiber-reinforced epoxy posts. (g) Fracture line visible after opening the flap. (h) Fracture line covered with MMA-based resin cement. (i) Periodontal tissue after surgery. (j) Radiograph after fixation and surgery. (k) Frontal view and (l) palatal view after composite resin filling of the palatal side. (m) Radiograph 3.5 years after intraoral cementation.

A 25-year-old female patient presented with a chief complaint of movement and pain during occlusion of the maxillary left central incisor. The labial gingiva was slightly swollen and reddened, and the labial tooth structure remained intact (Figure 2a). A dental periapical radiographic examination revealed that the traumatized tooth was devitalized and that the palatal cavity had been filled with composite resin without post material after root canal treatment (Figure 2b). Initial examination revealed a 6-mm periodontal pocket on the palatal-mesial side, along with Grade 1 tooth mobility. After clinical examination, the tooth structure was found to have fractured horizontally from the supramarginal palatal side to the submarginal labial side (Figure 2c). As an alternative, the extraction of the tooth and replacement using a fixed partial prosthesis or implant were presented; however, the patient strongly desired to preserve and restore the traumatized tooth and did not agree to any of the alternative treatment approaches. Thus, a treatment plan aiming to preserve the tooth was chosen.

A tight coronal seal of the root canal filling material was confirmed upon examination within the root canal; no problems were observed upon dental periapical radiographic examination. Furthermore, no clinical symptoms attributed to the root canal treatment were observed. Therefore, endodontic treatment was not considered. The fractured fragment was repaired using an MMA-based material (Super-Bond Opaque, Figure 2d). However, a sinus tract that penetrated the fractured part appeared on the labial gingiva (Figure 2e) 1 month after repair, indicating fracture reoccurrence at the bonded surface. Therefore, the abutment foundation was constructed using a glass fiber–reinforced epoxy post (i-TFC Luminous, three main points, each with a diameter of 1.0 mm). Moreover, a cylindrical subpoint called a sleeve was inserted to encase the central point. Another sleeve was divided vertically into two parts and inserted to fit along the root canal wall at the cervical part of the tooth (Figure 2f). Regenerative periodontal therapy was performed as in Case 1 (Figures 2g, 2h, and 2i). The fracture was visually confirmed to have occurred 2 mm above the alveolar bone margin by opening the flap. Appropriate repositioning was performed and confirmed by a postoperative dental radiograph (Figure 2j). The crown was then securely fixed to both adjacent teeth using clear-colored MMA-based resin cement (Super-Bond Clear).

The patient was monitored for 8 months posttreatment, and the stabilization of the periodontal tissue was confirmed. The labial tooth structure was left intact, and the palatal cavity was filled with a composite resin material to complete the treatment (Figure 2k,l). Approximately 3.5 years after the final treatment, the patient continues to maintain high esthetic outcomes, with no abnormalities on the dental radiographic image (Figure 2m).

## 3. Discussion

Horizontal root fractures are generally treated with conservative approaches, such as coronal fragment repositioning and splinting with the adjacent teeth; the success rate of conservative treatment without extraction is high [11]. However, root fractures occurring close to the bone margin, such as those reported in these two cases, reportedly have a poor prognosis [7].

When restoring a tooth with a horizontal root fracture near the buccal bone margin, minor surgery is effective in improving the condition of the periodontal tissue. However, if the fracture occurs in a central incisor, esthetics can deteriorate unless the balance with the periodontal tissue of the adjacent central incisor is taken into consideration. In this case series, minimal periodontal surgery was performed for horizontal root fractures near the bone margins.

No patients in this report had a labial ferrule. The periodontal tissue is prone to persistent inflammation in cases of loss of supragingival tooth structure labially, making the fabrication of fixed prostheses challenging. However, the presence of a small amount of palatal ferrule significantly improved the fracture resistance of the restored teeth [8].

To minimize damage due to the absence of a labial ferrule, multiple glass fiber–reinforced epoxy posts were placed on both the labial and palatal sides. A high fiber filling rate, especially near the cervical region, appeared to be beneficial for the integration of the remaining tooth structure with the prosthesis. Xiong et al. reported the effectiveness of fiber-reinforced posts wrapped in a tubular sleeve material in reinforcing a pulpless tooth with a flared root [19]. In Case 2, a unique method was employed that involved vertically splitting the sleeve. Vertical division allows the sleeve to deform easily and remain positioned along the wall of the root canal. The use of a sleeve is effective for abutment construction in unfavorable conditions because it can efficiently fill the root canal with fibers.

The foundation method using multiple fiber posts is quite distinctive. Although no clinical evidence exists to indicate the influence of the number or position of fiber posts for foundations, using composite foundation materials with higher filler content, including fibers, significantly improved fracture resistance compared with the use of conventional composite resins [14]. In fact, almost all of the restorations in our clinics using the same fiber arrangement method introduced in this study have demonstrated good clinical outcomes without any complications, such as fractures or detachments, to date.

Thus, even if restorations were placed in clinical practice using the procedure described in this study, the bonded part may not adhere properly, causing refracture or reinfection, resulting periodontal tissue destruction. If such problems occur in the future, re-restoration or tooth extraction may be warranted. However, dental trauma rates are reportedly much higher in children than in adults. As a result of these injuries, oblique root fractures are also encountered. Pedodontists often recommend maintaining such teeth in the mouth until the age when fixed partial prostheses or implants can be considered, despite their poor prognosis. The technique described in this report may be useful in treating such cases in which immediate extraction is not recommended.

This study reports the treatment of two adult cases; other general prosthetic methods such as fixed partial prosthesis or implant could have been selected. A conservative method was chosen due to the strong wishes of the patients, warranting a lengthy treatment period. The risk of refracture cannot be denied. It should be noted that this method is not necessarily acceptable to and recommended by all clinicians, which is a significant limitation of the study.

The adhesive interface remained in the periodontal pocket even after surgery and restorative treatment because the fracture occurred near the alveolar bone margin. Therefore, the cement materials used should exhibit excellent biocompatibility. In addition, the flexibility of the foundation material helps prevent refracture of the fractured roots. Therefore, an MMA-based nonfiller cement was selected as the foundation material. The appropriate selection of materials, such as restorative and cement materials, and the adequate choice of surgical techniques are important factors for the long-term survival of fractured teeth. In the future, further in vitro investigations are required, especially on the multiple and mixed-size fiber arrangement and the core resin material that was employed in the present study. In addition, objective evaluation of the longevity of the treatments performed in these cases via statistical analyses would benefit this report.

In conclusion, root fractures near the alveolar bone margin generally have a poor prognosis. Nonetheless, the outcomes of the two cases presented herein suggest that esthetic restoration can be achieved with the use of the appropriate surgical techniques and the selection of foundation materials, without orthodontic treatment.

## Data Availability

The data that support the findings of this study are available on request from the corresponding author. The data are not publicly available due to privacy or ethical restrictions.

## References

[1] Prithviraj D. R., Bhalla H. K., Vashisht R., Regish K. M., Suresh P. (2014). An Overview of Management of Root Fractures. *Kathmandu University Medical Journal*.

[2] Feiglin B. (1995). Clinical Management of Transverse Root Fractures. *Dental Clinics of North America*.

[3] Malhotra N., Kundabala M., Acharaya S. (2011). A Review of Root Fractures: Diagnosis, Treatment and Prognosis. *Dental Update*.

[4] Ranka M., Shah J., Youngson C. (2012). Root Fracture and Its Management. *Dental Update*.

[5] Erdem A. P., Ozdas D. O., Dincol E., Sepet E., Aren G. (2009). Root healıng wıth MTA After horıizontal Fracture. *European Archives of Paediatric Dentistry*.

[6] Mane N. A., Shetty P., Borkar A. C., Mujumdar S. V., Mujawar A. (2023). Healing After Horizontal Root Fracture of Maxillary Central Incisor: A Case Report With 24-Month Follow-Up. *Cureus*.

[7] Welbury R., Kinirons M. J., Day P., Humphreys K., Gregg T. A. (2002). Outcomes for Root-Fractured Permanent Incisors: A Retrospective Study. *Pediatric Dentistry*.

[8] Zhang Y. Y., Peng M. D., Wang Y. N., Li Q. (2015). The Effects of Ferrule Configuration on the Anti-Fracture Ability of Fiber Post-Restored Teeth. *Journal of Dentistry*.

[9] Lazari P. C., de Carvalho M. A., Del Bel Cury A. A., Magne P. (2018). Survival of Extensively Damaged Endodontically Treated Incisors Restored With Different Types of Posts-and-Core Foundation Restoration Material. *Journal of Prosthetic Dentistry*.

[10] Hovland E. J. (1992). Horizontal Root Fractures. *Dental Clinics of North America*.

[11] Lo Giudice R., Lizio A., Cervino G. (2018). The Horizontal Root Fractures. Diagnosis, Clinical Management and Three-Year Follow-Up. *Dental Journal*.

[12] Marzadori M., Stefanini M., Sangiorgi M., Mounssif I., Monaco C., Zucchelli G. (2018). Crown Lengthening and Restorative Procedures in the Esthetic Zone. *Periodontology 2000*.

[13] Thoma D. S., Benić G. I., Zwahlen M., Hämmerle C. H., Jung R. E. (2009). A Systematic Review Assessing Soft Tissue Augmentation Techniques. *Clinical Oral Implants Research*.

[14] Shah E. H., Shetty P., Aggarwal S., Sawant S., Shinde R., Bhol R. (2021). Effect of Fibre-Reinforced Composite as a Post-Obturation Restorative Material on Fracture Resistance of Endodontically Treated Teeth: A Systematic Review. *Saudi Dental Journal*.

[15] Fujisawa S., Atsumi T. (2004). Cytotoxicities of a 4-META/MMA-TBBO Resin Against Human Pulp Fibroblasts. *Dental Materials Journal*.

[16] Garza E. G., Wadajkar A., Ahn C. (2012). Cytotoxicity Evaluation of Methacrylate-Based Resins for Clinical Endodontics In Vitro. *Journal of Oral Science*.

[17] Suzuki R., Kuroyanagi Y. (2013). Safety and Utility of a PMMA-Based Tissue Adhesive for Closure of Surgical Incision Wounds. *Journal of Biomaterials Science. Polymer Edition*.

[18] Tsuchiya Y., Muramatsu T., Masaoka T., Hashimoto S., Shimono M. (2009). Effect of the Dental Adhesive, 4-META/MMA-TBB Resin, on Adhesion and Keratinization of Regenerating Oral Epithelium. *Journal of Periodontal Research*.

[19] Xiong Y., Huang S. H., Shinno Y. (2015). The Use of a Fiber Sleeve to Improve Fracture Strength of Pulpless Teeth With Flared Root Canals. *Dental Materials*.

[20] Yoshii S., Shimizu H., Nishino T. (2016). The Bending Strength and Durability of Fiber Post and Core Systems Using a Sleeve. *Japanese Journal of Conservative Dentistry*.

